# The Initial Relationship Between the United States Department of Health and Human Services’ Digital COVID-19 Public Education Campaign and Vaccine Uptake: Campaign Effectiveness Evaluation

**DOI:** 10.2196/43873

**Published:** 2023-05-03

**Authors:** Christopher J Williams, Elissa C Kranzler, Joseph N Luchman, Benjamin Denison, Sean Fischer, Thomas Wonder, Ronne Ostby, Monica Vines, Jessica Weinberg, Elizabeth L Petrun Sayers, Allison N Kurti, Sarah Trigger, Leah Hoffman, Joshua F A Peck

**Affiliations:** 1 Fors Marsh Arlington, VA United States; 2 US Department of Health and Human Services Office of the Assistant Secretary for Public Affairs Washington, DC United States

**Keywords:** communication campaign, COVID-19, COVID-19 pandemic, COVID-19 vaccination, public education campaign, public health campaign, social marketing, marketing, campaign, vaccination, patient education, United States, vaccine, digital impression, public education

## Abstract

**Background:**

Over 1 million people in the United States have died of COVID-19. In response to this public health crisis, the US Department of Health and Human Services launched the *We Can Do This* public education campaign in April 2021 to increase vaccine confidence. The campaign uses a mix of digital, television, print, radio, and out-of-home channels to reach target audiences. However, the impact of this campaign on vaccine uptake has not yet been assessed.

**Objective:**

We aimed to address this gap by assessing the association between the *We Can Do This* COVID-19 public education campaign’s digital impressions and the likelihood of first-dose COVID-19 vaccination among US adults.

**Methods:**

A nationally representative sample of 3642 adults recruited from a US probability panel was surveyed over 3 waves (wave 1: January to February 2021; wave 2: May to June 2021; and wave 3: September to November 2021) regarding COVID-19 vaccination, vaccine confidence, and sociodemographics. Survey data were merged with weekly paid digital campaign impressions delivered to each respondent’s media market (designated market area [DMA]) during that period. The unit of analysis was the survey respondent–broadcast week, with respondents nested by DMA. Data were analyzed using a multilevel logit model with varying intercepts by DMA and time-fixed effects.

**Results:**

The *We Can Do This* digital campaign was successful in encouraging first-dose COVID-19 vaccination. The findings were robust to multiple modeling specifications, with the independent effect of the change in the campaign’s digital dose remaining practically unchanged across all models. Increases in DMA-level paid digital campaign impressions in a given week from –30,000 to 30,000 increased the likelihood of first-dose COVID-19 vaccination by 125%.

**Conclusions:**

Results from this study provide initial evidence of the *We Can Do This* campaign’s digital impact on vaccine uptake. The size and length of the Department of Health and Human Services *We Can Do This* public education campaign make it uniquely situated to examine the impact of a digital campaign on COVID-19 vaccination, which may help inform future vaccine communication efforts and broader public education efforts. These findings suggest that campaign digital dose is positively associated with COVID-19 vaccination uptake among US adults; future research assessing campaign impact on reduced COVID-19–attributed morbidity and mortality and other benefits is recommended. This study indicates that digital channels have played an important role in the COVID-19 pandemic response. Digital outreach may be integral in addressing future pandemics and could even play a role in addressing nonpandemic public health crises.

## Introduction

The COVID-19 pandemic has led to more than 104 million COVID-19 cases and over 1 million COVID-19 deaths in the United States as of April 3, 2023 [1]. COVID-19 vaccines are safe and effective [2] and are estimated to have prevented more than 66 million COVID-19 cases and 17 million COVID-19–related hospitalizations, saving more than 2 million lives and almost US $900 billion in health care costs in the United States from mid-December 2020 through the end of March 2022 [3]. Vaccination research predating the COVID-19 pandemic has demonstrated that it is necessary not only to make vaccines available and accessible but also to address vaccine hesitancy, which is the “delay in acceptance or refusal of vaccination despite the availability of vaccination services” [4].

Vaccine hesitancy is a critical barrier to vaccine uptake [5], suggesting that interventions that aim to decrease vaccine hesitancy may result in increased vaccine uptake. In the context of COVID-19, research has shown that vaccine hesitancy predicts vaccine uptake [6], such that groups of individuals who were initially less vaccine-hesitant were more likely to report subsequent vaccination. Other research suggests that widespread COVID-19 vaccine uptake requires the application of multicomponent interventions that raise knowledge and awareness to address vaccine hesitancy and influence behavior change [7,8]. Between April 2021 and April 2022, nearly 148.6 million people received a first-dose COVID-19 vaccination in the United States [5].

Public education campaigns, which reach and engage large population segments through a mix of media channels, have demonstrated a measurable impact on a range of health behaviors [9,10] and have successfully influenced vaccine hesitancy and vaccine uptake in other contexts [7]. In response to the COVID-19 pandemic, the US Department of Health and Human Services (HHS) launched the *We Can Do This* public education campaign (the campaign) [11] in April 2021 to increase COVID-19 vaccine confidence (the likelihood of vaccination) and, ultimately, vaccine uptake. (In December 2020, the HHS launched the *Slow the Spread* campaign, which encouraged mask wearing and social distancing.)

The *We Can Do This* campaign aims to influence COVID-19 vaccine confidence and uptake through the dissemination of advertisements (eg, 30-second videos and static images with text) that address key attitudinal and behavioral constructs relevant to these outcomes across a mix of traditional and new media channels. These channels include television, radio, and print media; site direct (digital advertising directly purchased on websites), programmatic (digital advertising purchased through automated marketplace platforms to reach audiences across a range of websites, apps, and platforms), and paid social media (advertising bought directly on social media platforms) advertisements; earned media; partnerships; and influencer engagement. To reach diverse audiences, the campaign has engaged simultaneously with the general population and with specific racial and ethnic audiences through tailored communications in more than 14 languages, including English and Spanish.

Between April 5 and September 26, 2021, according to Nielsen Digital and Total Ad Ratings (see Multimedia Appendix 1), the campaign is estimated to have reached more than 90% of US adults an average of 20.9 times across measured television and digital channels (Nielsen Digital Ad Ratings, unpublished data, 2021). In addition to the campaign’s national reach, it also delivered extra ads to markets, zip codes, and population segments with higher proportions of vaccine-hesitant adults and higher COVID-19 prevalence. As the vaccination uptake rate varied across designated market areas (DMAs), the campaign also took vaccination rates into account when deciding where to deliver these extra ads to help encourage first-dose vaccination.

To date, there have been no published evaluations of the impact of this campaign on COVID-19 vaccine uptake. This study is the first to assess the association between digital campaign media dose—an under-studied avenue for public education campaign dissemination—and an individual’s likelihood of receiving their first COVID-19 vaccination dose.

## Methods

### Overview

To evaluate the potential association between the campaign and vaccine uptake, we used individual-level survey data and market-level campaign media dose data. Digital campaign media dose refers to the aggregation of all digital ads that were placed in a DMA at a given time. The individual-level data were derived from the COVID-19 Attitudes and Beliefs Survey (CABS), a nationally representative, probability-based longitudinal survey of US adults (aged 18+ years) administered every 4 months to the same individuals through the AmeriSpeak probability-based research panel of the National Opinion Research Center (NORC) [12]. The 35-minute web-based survey measures adherence to COVID-19 preventive behaviors, including COVID-19 vaccination, and respondents’ sociodemographic characteristics. Analysis was conducted with data from the 3642 respondents who completed survey waves 1-3, as wave 3 was the first wave in which respondents provided the date of their first COVID-19 vaccination. (Details about survey administration and a table of unweighted descriptive statistics are included in Multimedia Appendix 1.) Paid campaign media dose data were collected for digital platforms (ie, site direct, social media, and programmatic advertisements; described in Multimedia Appendix 1) from campaign launch on April 1 to November 7, 2021, the last date of CABS wave 3 completion. This represents all the paid digital media dose administered during this period as part of the campaign.

### Ethics Approval

We sought institutional review board (IRB) approval for this study from the Biomedical Research Alliance of New York, an external IRB service accredited by the Association for the Accreditation of Human Research Protection Programs. The study protocol and materials were reviewed and approved by Biomedical Research Alliance of New York’s social and behavioral IRB (Federalwide Assurance FWA00000337, protocol 20-077-821).

### Informed Consent, Respondent, Confidentiality, and Compensation

Although all respondents provided consent as part of their registration into their associated panel, we ensured that all qualifying respondents provided informed consent to participate in the study. The consent language was available on the web, programmed into the final part of the screener. After screening respondents, we directed those eligible (ie, respondents who did not screen out) to read the consent language. If they decided to participate, eligible respondents electronically provided consent and were directed to the web-based survey. Although this study presented minimal risk of harm to subjects, all respondents were informed at the beginning of the survey that any questions that make them feel uncomfortable may be skipped or ignored. We included links to mental health resources for respondents to access if they experienced any distress from participating in the study.

To ensure respondent confidentiality, (1) data transfer was conducted via a secure, password-protected site; (2) all screening-related information was not tied to any personal identifiable information, but identified and matched by the assigned unique ID; (3) data sets and reports did not contain any personal identifiable information; and (4) respondents were not tied to individual responses, and any data used in reporting were not be attributed to specific respondents. Data were tightly controlled behind firewalls with password-protected access by senior researchers. All final data were stored in a secure environment that does not have access to the internet and requires a separate access code by researchers. Researchers were trained to never export data from this secure server.

Respondents who decided to participate were offered US $10 in the first wave of the CABS and US $18 for each subsequent wave of the survey.

### Measures

#### Dependent Variable

The dependent variable was dichotomous, indicating whether a respondent reported receiving the first dose of a COVID-19 vaccination in each broadcast week. The unit of analysis was the respondent-broadcast week; we used broadcast weeks, which run from Monday to Sunday, because that is how advertising is purchased. Within the data set, there was an observation for each CABS respondent in each broadcast week starting the week of November 30, 2020, as this date marks the beginning of the first broadcast week in which a vaccine was publicly available. If a respondent did not report having been vaccinated in a broadcast week, then they were included as an observation in the subsequent broadcast week. If a respondent reported having been vaccinated in a broadcast week, then they were not included as an observation in the subsequent broadcast week. Some respondents (n=241) reported vaccination dates that occurred before the date of the US Food and Drug Administration (FDA) emergency use authorization (EUA). Under the assumption that these individuals misstated the year of vaccination, it was changed from 2020 to 2021 in these instances. As a robustness test, we conducted analyses in which individuals who reported a vaccination date before the FDA EUA were dropped. As the results were similar, we retained them in the analysis.

#### Independent Variable

The independent variable was paid campaign digital media dose, representing the change in the total number of site direct, programmatic, and social media advertisement impressions (impressions are the digital publishers’ estimates of the number of times an advertisement is seen or heard) in a DMA (a DMA region is a group of counties and zip codes that form an exclusive geographic area in which the home market television stations are the predominant stations in terms of total hours viewed; DMA is a proprietary construct of the Nielsen Company) per 100,000 people between *week_t-2_* and *week_t-1_*. This operationalization allowed us to assess the short-term relationship between increasing digital media dose in a DMA and the individual-level likelihood of first-dose vaccination. We used the change in impressions between *week_t-2_* and *week_t-1_* because we expected a lag between one’s decision to get vaccinated and vaccine receipt due to logistics (eg, navigating appointment availability and scheduling), such that adding or decreasing campaign dose would not immediately influence vaccinations. The change in dose in each DMA reflects that the campaign varied the distribution of advertising over both week and markets; therefore, markets had a lower, same, or higher dose of advertising from week to week. Respondents were assigned a digital dose by broadcast week based on their DMA of residence.

#### Covariates

To account for the potential influence of factors exogenous to the campaign that could still be correlated with changes in media dose, analyses controlled for the change in weekly COVID-19 cases and deaths and the change in weekly cable news COVID-19 coverage by DMA between *week_t-2_* and *week_t-1_*. Cable news is used as a measure of COVID-19 salience, and although this is only one form of media, it can serve as a proxy for all media discussion of the topic due to the intermedia agenda-setting effect [13]. To account for the potential influence of sociodemographic characteristics on first-dose vaccination, we controlled for respondent age, sex, race/ethnicity, education, household income, political ideology, rurality, essential worker status, and preexisting health conditions as reported in CABS wave 3. A model accounting for whether an individual is insured is included in Multimedia Appendix 1. The results for the main independent variable are nearly identical; however, we chose not to include this variable in the main model as it is correlated with other demographic variables.

We expected that individuals may have predispositions that may influence the effectiveness of the campaign and their likelihood to get a vaccination, so we controlled for respondent vaccine confidence (ie, a respondent’s reported vaccine uptake or likelihood that they will get vaccinated against COVID-19) as reported in CABS wave 1. Details about COVID-19 cases and deaths data, COVID-19 cable news coverage data, sociodemographic variables, and vaccine confidence, including a discussion of the coding of these variables, are provided in Multimedia Appendix 1.

### Statistical Analysis

**Figure 1:**
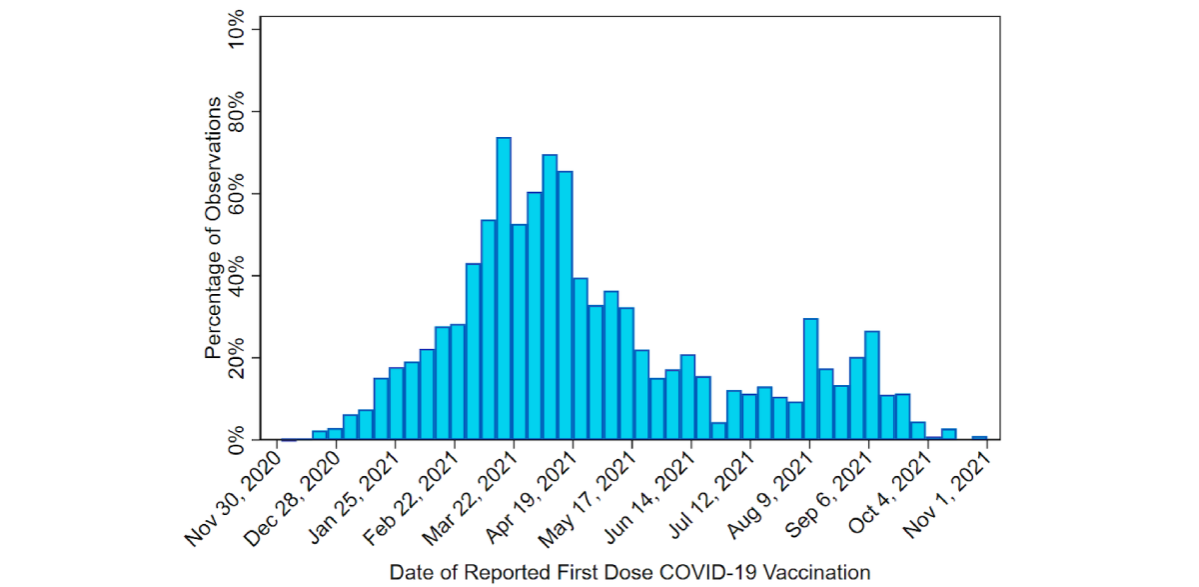
Histogram of reported first dose of COVID-19 vaccination dates, United States, December 1, 2020, to November 7, 2021.

Before conducting analyses, we examined the independent and dependent variable distributions to inform our analytic approach. The earliest a respondent reported receiving the first dose of a COVID-19 vaccine was December 2, 2020 (see Multimedia Appendix 1 for a discussion of robustness tests relevant to vaccination date). Figure 1 presents a histogram of the dates that respondents reported first-dose vaccination. The largest percentage of first-dose vaccinations occurred in March 2021, with the first-dose vaccination rate dropping steadily through April to July 2021. First-dose vaccination increased slightly in August 2021 before dropping off substantially in October 2021. If an individual was not vaccinated by the date on which they completed CABS wave 3, then their last observation in the data set was the broadcast week of their wave 3 survey completion date.

The change in digital media dose by DMA, the main independent variable, ranged from –155,716.4 to 117,041.3, with a mean of 520.6 (SD 14, 281.69). Table S1 in Multimedia Appendix 1 presents descriptive statistics for all variables in the analysis.

To assess the relationship between digital media dose and first-dose COVID-19 vaccination, we estimated a series of multilevel logistic regression models, with varying intercepts by DMA to account for the nesting of respondents within DMAs. The SEs of these models are clustered by DMA. The intraclass correlation coefficient for the main model was 0.03, indicating that about 3% of the variance in the outcome variable varies across DMAs.

We estimated 4 regression models in a stepwise manner. Model 1 (baseline model) estimates the relationship between change in digital media dose between *week_t-2_* and *week_t-1_* and the likelihood of first-dose COVID-19 vaccination. In model 2, we estimated the baseline model, including controls for exogenous COVID-19–relevant factors. In model 3, we also controlled for the sociodemographic characteristics of survey respondents. In model 4, we included an additional control for respondents’ vaccine confidence as reported in wave 1. As there may have been certain time periods in which a DMA was more likely to see changes in digital impressions and individuals were more likely to receive a vaccination, we included week dummy variables in all models. All models were weighted and design adjusted (see Survey Weighting in Multimedia Appendix 1). Analyses were conducted using Stata (version 17; StataCorp) [14]. Multimedia Appendix 1 includes a discussion of the regression model specification for the primary model below (model 4).

## Results

### Relationship Between Digital Advertising Media Dose and the Likelihood of Vaccination

Table 1 presents results from all regression models. As the models used in this study are multilevel logit models, the coefficients cannot be directly interpreted in terms of substantive effect. Rather, we calculate marginal effects (see below) to understand the substantive effects of each variable.

Results for model 1 show a positive and statistically significant relationship between the weekly change in digital impressions and the likelihood of first-dose vaccination (β=.000014; Z=3.22; *P*=.001), indicating that an increase in digital impressions within a DMA was associated with a higher likelihood of a respondent in that DMA reporting having received a first-dose COVID-19 vaccination in the subsequent week.

There were no substantive differences between models 1 and 2 in the effects of change in digital media dose on the likelihood of first-dose COVID‑19 vaccination; the effect on the likelihood of first-dose vaccination continued to be positive (β=.000014; Z=3.16; *P*=.002) after controlling for factors exogenous to the campaign. The change in new COVID-19 cases was negative and significant (β_cases_=–.0006; Z=–1.99; *P*=.047), whereas the change in deaths was negative and insignificant (β_deaths_=–.01; Z=–1.18; *P*=.24). The total change in COVID-19 cable news coverage was positive and insignificant (β=.172; Z=0.24; *P*=.81). (The week dummy variable format makes it difficult to fully assess the effect of COVID-19 cable news coverage.)

**Table 1:** Relationship between digital advertising media dose and the likelihood of vaccination, United States, December 1, 2020, to November 7, 2021^a^.

			Model 1					Model 2				Model 3				Model 4		
			Value		*P* value		Value		*P* value		Value			*P* value	Value			*P* value
Δ HHS^b^ digital impressions, β (SE)			0.000014 (0.000004)		.001		0.000014 (0.000004)		.002		0.000013 (0.000004)			.002	0.000014 (0.0000004)			.002
**Exogenous factors, β (SE)**																		
	Δ COVID-19 cases	—^c^		—		–0.0006 (0.0003)			.047	–0.0006 (0.0003)			.04		–0.0006 (0.0003)		.051	
	Δ COVID-19 deaths	—		—		–0.0105 (0.0090)			.24	–0.0069 (0.0085)			.42		–0.0073 (0.0084)		.38	
	Δ COVID-19 cable news coverage	—		—		0.1720 (0.7057)			.81	0.1670 (0.7201)			.81		0.1515 (0.7297)		.84	
**Demographics, β (SE)**																		
	Income	—		—		—			—	0.169 (0.022)			<.001		0.115 (0.028)		<.001	
	Female sex	—		—		—			—	–0.114 (0.051)			.03		–0.010 (0.06)		.88	
	Age	—		—		—			—	0.456 (0.04)			<.001		0.378 (0.04)		<.001	
	Education	—		—		—			—	0.219 (0.03)			<.001		0.169 (0.04)		<.001	
	Essential worker status	—		—		—			—	–0.101 (0.07)			.13		–0.060 (0.08)		.47	
	Political ideology	—		—		—			—	–0.448 (0.036)			<.001		–0.245 (0.043)		<.001	
	Preexisting health condition	—		—		—			—	0.191 (0.055)			.001		0.099 (0.055)		.08	
	Rurality	—		—		—			—	–0.118 (0.046)			.01		–0.095 (0.049)		.05	
	Black or African American race	—		—		—			—	–0.170 (0.085)			.04		0.035 (0.076)		.64	
	Hispanic/Latino ethnicity	—		—		—			—	0.154 (0.073)			.04		0.177 (0.075)		.02	
**Initial vaccine confidence, β (SE)**																		
	Wave 1 Vaccine Confidence	—		—		—			—	—			—		1.070 (0.058)		<.001	
**Week dummy variables not reported for brevity**																		
	Constant, β (SE)	–3.5199 (1.068)		.001		–2.240 (4.449)			.62	–2.637 (4.555)			.56		–5.0876 (4.639)		.27	
	DMA^d^ variance, β (SE)	0.103 (0.024)		—		0.100 (0.024)			—	0.090 (0.022)			—		0.111 (0.029)		—	
	Observations, n	76,128		—		76,128			—	76,128			—		76,128		—	
	DMAs, n	204		—		204			—	204			—		204		—	

^a^The dependent variable is a dichotomous measure of whether a respondent received the first dose of a COVID-19 vaccine in each week.

^b^HHS: Department of Health and Human Services.

^c^Not applicable.

^d^DMA: designated market area.

The relationship between change in digital media dose and the likelihood of first-dose vaccination was positive and statistically significant for both models 3 and 4 (β_model3_=.000013; Z=3.12; *P*=.002 and β_model4_=.000014; Z=3.13; *P*=.002, respectively) after controlling for respondents’ sociodemographic characteristics and vaccine confidence. In fact, there was a minimum difference seen in the effect of the change in digital media dose when controlling for sociodemographics. This indicates that individual sociodemographics do not change the overall effect of the campaign, which is as expected given that any one individual’s age, income, ideology, etc, is in all likelihood not correlated with the campaign’s decision to increase or decrease digital dose in the DMA in which that individual lives.

Taken together, models 1-4 consistently indicated that an increase in the number of digital impressions in a DMA between *week_t-2_* and *week_t-1_* was associated with an increased likelihood that an individual in that DMA received their first dose of a COVID-19 vaccine in the subsequent week.

### Substantive Effects of Digital Dose on the Likelihood of Vaccination

**Figure 2:**
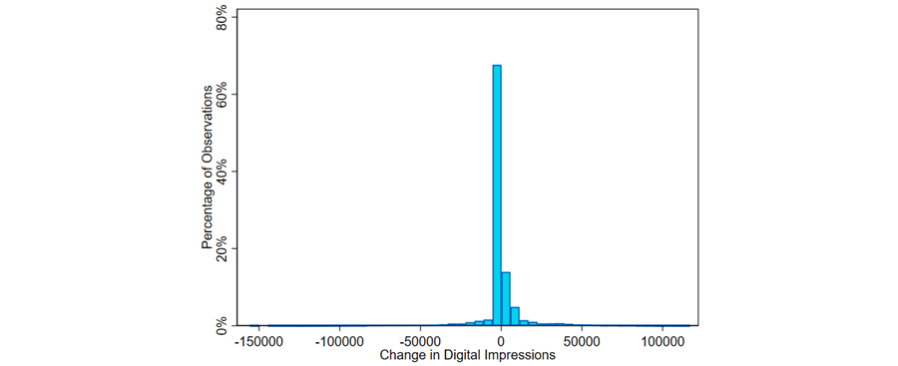
The distribution of change in digital impressions, United States, April 1, 2021, to November 7, 2021.

To examine the substantive relationship between the change in digital campaign impressions and the likelihood of first-dose COVID-19 vaccination, we estimated the expected probability of first-dose vaccination across levels of weekly change in digital impressions while holding all other variables at their means. More than 95% of all observations fell between –30,000 and 30,000 impressions, although this variable ranged from about –155,000 to a high of about 117,000 in some markets (Figure 2).

**Figure 3:**
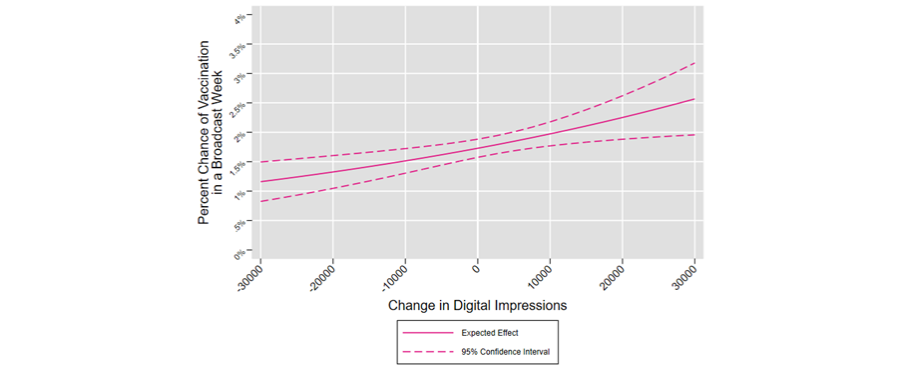
Average change in weekly digital impressions on the likelihood of individual first-dose vaccination, United States, December 1, 2020, to November 7, 2021.

Figure 3 illustrates the expected probability of first-dose vaccination as a function of the change in digital media dose when this variable falls between –30,000 and 30,000 impressions. The solid pink line is the expected probability of an individual receiving a first-dose vaccine, given the change in digital impressions in their DMA in the previous week, while holding all other variables at their means. The dashed lines represent the 95% CI.

When digital impressions in a DMA decreased by 30,000 between *week_t-1_* and *week_t-2_*, the chance of a respondent in that DMA receiving a first-dose COVID-19 vaccine in *week_t-0_* was about 1.2%. When digital impressions did not change between *week_t-1_* and *week_t-2_*, the chance of a respondent receiving the first dose of a COVID-19 vaccine was one-third greater (1.7%). When digital impressions increased by 30,000 between *week_t-1_* and *week_t-2_*, the chance of a respondent receiving a first-dose COVID-19 vaccine was 2.6%. In other words, increasing digital impressions from 0 to 30,000 in a given week increased the likelihood of being vaccinated by 53%. Increasing digital impressions from –30,000 to 30,000 in a given week more than doubled (125% increase) the likelihood of being vaccinated. The average marginal effect on the likelihood of receiving a first-dose COVID-19 vaccination in a broadcast week given a change of 1 additional impression is 0.0000433%.

## Discussion

### Principal Findings

This study assessed the relationship between paid campaign digital media and the likelihood of COVID-19 vaccination in a representative sample of US adults. Results demonstrate a positive and significant relationship between the weekly change in digital impressions and the likelihood of first-dose vaccination, providing initial evidence that the digital campaign has been effective in increasing COVID-19 vaccination among US adults. This association remained statistically significant after controlling for a series of covariates, including COVID-19 cases and deaths as well as respondents’ sociodemographic characteristics and baseline vaccine confidence, indicating that results are robust to the inclusion of other factors. It is possible that the association is a factor of both getting people who would otherwise not be vaccinated to do so and shortening the time to vaccination, both of which are of substantial importance during a pandemic. Future research could use event-history modeling to explore these possibilities.

Results indicate that when change in digital impression exposure is held at 0, older respondents compared to younger respondents, those with higher incomes compared to those with lower incomes, and those with higher education compared to those with lower levels of education were significantly more likely to receive first-dose COVID-19 vaccination. These findings, which are independent of the campaign’s effects, align with recent research demonstrating that willingness to vaccinate is higher among individuals aged 65 years and older compared to younger groups [15]; that lower income individuals are less willing to get vaccinated compared to those with higher incomes [16]; and, relative to individuals with less education, those with a college degree or higher reported greater vaccine acceptance [17]. Results also demonstrate that, independent of digital campaign exposure, politically conservative respondents were significantly less likely to be vaccinated compared to more liberal respondents, echoing findings from other research that show political conservatism is negatively associated with intentions to get vaccinated and vaccine uptake [18,19].

Importantly, much of the extant literature examines longer-term effects (eg, recalled campaign exposure or campaign impressions aggregated over longer periods of time) on behavior change [20-22], whereas this study examines the relationship between weekly change in digital impressions and vaccination uptake. Focusing on the short-term effects of digital dose may represent a more conservative approach, likely underestimating the total effect of the digital campaign on COVID-19 vaccination. Further, this study is somewhat limited in scope, as it focuses only on the relationship between the digital campaign and vaccine uptake and does not account for additional media channels through which the campaign was disseminated (eg, television and out-of-home advertising), all of which may impact the relationship between the campaign and vaccine uptake. Although this is a first assessment of the impact of digital impressions on COVID-19 vaccine uptake, a fruitful avenue for future research may involve the incorporation of other media channels through which the campaign was disseminated, to test the association between campaign impressions and vaccinations more comprehensively.

### Limitations

This study’s results reflect a discrete period and single media channel and may not reflect the influence of the campaign on the likelihood of vaccination during other time periods or channels through which the campaign has been disseminated (eg, print and radio). The recalled date of first-dose vaccination was subject to recall bias and may not reflect respondents’ actual date of vaccination. All impressions data were aggregated by DMA; however, the dependent variable was provided at the respondent level. Weekly changes in media dose by DMA functioned as a measure of probable dose, exogenous to our survey data, but does not represent confirmed campaign exposure among respondents. It is possible that weighting methodologies could influence findings; however, sensitivity checks found little change in the association (Multimedia Appendix 1). Previous evaluations [20-22] have examined effects of campaign exposure aggregated over longer periods, whereas this study examined short-term effects (ie, week-over-week change) of the campaign on vaccine uptake. This is likely a conservative approach, which could lead to an underestimation of campaign effects.

Although our models included potential influencing factors for vaccine uptake, the variable list was not exhaustive, and analyses may have been subject to the influence of unmeasured confounders. For more than 2 years, US adults have been exposed to information and conversations about COVID-19 vaccination from government sources (eg, federal agencies and state and municipal health departments), health care representatives (eg, health care professionals and pharmaceutical companies), community-based organizations, and friends and family. Concurrently, many government, travel, and employer vaccination mandates and policies were implemented during the study period. It is possible that first-dose vaccination in our study sample was influenced by one or several other factors not included in our models. Further, the change in the campaign’s digital dose may have differential effects based on geography, with the campaign being more successful in certain regions of the country. This may be an interesting area for future research.

### Conclusions

The COVID-19 pandemic represents one of the largest public health crises of our era [23]. Public education campaigns can help promote COVID-19 vaccine uptake [24]. Results from this study offer the first evidence of a large-scale digital COVID-19 public education campaign’s initial impact on vaccine uptake. The size and length of the HHS COVID-19 *We Can Do This* public education campaign make it uniquely situated to examine the impact of a digital campaign on COVID-19 vaccination, which may help inform future vaccine communication efforts and broader public education efforts. These findings suggest that campaign digital dose has attenuated the burden of COVID-19 in the United States; future research may be useful in assessing campaign impact on reduced COVID-19–attributed morbidity and mortality and other benefits.

### Public Health Implications

This study’s findings show that the HHS COVID-19 public education campaign was associated with a greater likelihood of individual vaccination in any given week during the period of April 1 to November 7, 2021. People who reported living in areas with more digital campaign impressions were more likely to be vaccinated, as increasing digital impressions from –30,000 to 30,000 in a given week more than doubled (125% increase) the likelihood of being vaccinated. These findings indicate that, similar to public education campaign influence on other health behaviors [9,10], the HHS COVID-19 public education campaign has played a key role in influencing COVID-19 vaccine uptake in the United States. Public education campaigns have promise to influence other COVID-19 vaccination behaviors, such as encouraging parents to get their eligible children vaccinated and encouraging eligible adults to get a COVID-19 booster.

## Appendix

Multimedia Appendix 1 Additional methods, Nielsen Digital Ad Ratings, and supplementary tables.

## Abbreviations

CABS: COVID-19 Attitudes and Beliefs Survey
DMA: designated market area
EUA: emergency use authorization
FDA: Food and Drug Administration
HHS: Department of Health and Human Services
IRB: institutional review board
NORC: National Opinion Research Center

## Data Availability

Data used for this analysis are currently not available due to permissions.

## References

[1] (2022). COVID Data Tracker. Centers for Disease Control and Prevention.

[2] (2021). COVID-19 Vaccination. Centers for Disease Control and Prevention.

[3] Schneider EC, Shah A, Sah P, Vilches T, Pandey A, Moghadas S, Galvani AP (2022). Impact of U.S COVID-19 vaccination efforts: an update on averted deaths, hospitalizations, and health care costs through March 2022. The Commonwealth Fund.

[4] MacDonald NE, SAGE Working Group on Vaccine Hesitancy (2015). Vaccine hesitancy: definition, scope and determinants. Vaccine.

[5] Aw J, Seng JJB, Seah SSY, Low LL (2021). COVID-19 vaccine hesitancy-a scoping review of literature in high-income countries. Vaccines (Basel).

[6] Wagner AL, Porth JM, Wu Z, Boulton ML, Finlay JM, Kobayashi LC (2022). Vaccine hesitancy during the COVID-19 pandemic: a latent class analysis of middle-aged and older US adults. J Community Health.

[7] Jarrett C, Wilson R, O'Leary M, Eckersberger E, Larson HJ, SAGE Working Group on Vaccine Hesitancy (2015). Strategies for addressing vaccine hesitancy - a systematic review. Vaccine.

[8] Finney Rutten LJ, Zhu X, Leppin AL, Ridgeway JL, Swift MD, Griffin JM, St Sauver JL, Virk A, Jacobson RM (2021). Evidence-based strategies for clinical organizations to address COVID-19 vaccine hesitancy. Mayo Clin Proc.

[9] Wakefield MA, Loken B, Hornik RC (2010). Use of mass media campaigns to change health behaviour. Lancet.

[10] Anker AE, Feeley TH, McCracken B, Lagoe CA (2016). Measuring the effectiveness of mass-mediated health campaigns through meta-analysis. J Health Commun.

[11] Weber MA, Backer TE, Brubach A (2022). Creating the HHS COVID-19 public education media campaign: applying systems change learnings. J Health Commun.

[12] (2022). Technical overview of the AmeriSpeak® panel NORC's probability-based household panel. NORC.

[13] Vu HT, Guo L, McCombs ME (2014). Exploring “the World Outside and the Pictures in Our Heads”. Journal & Mass Commun Q.

[14] Stata Statistical Software: Release 17. StataCorp.

[15] Kelly BJ, Southwell BG, McCormack LA, Bann CM, MacDonald PDM, Frasier AM, Bevc CA, Brewer NT, Squiers LB (2021). Predictors of willingness to get a COVID-19 vaccine in the U.S. BMC Infect Dis.

[16] El-Mohandes A, White TM, Wyka K, Rauh L, Rabin K, Kimball SH, Ratzan SC, Lazarus JV (2021). COVID-19 vaccine acceptance among adults in four major US metropolitan areas and nationwide. Sci Rep.

[17] Yasmin F, Najeeb H, Moeed A, Naeem U, Asghar M, Chughtai N, Yousaf Z, Seboka B, Ullah I, Lin C, Pakpour A (2021). COVID-19 vaccine hesitancy in the United States: a systematic review. Front Public Health.

[18] Latkin C, Dayton L, Miller J, Yi G, Balaban A, Boodram B, Uzzi M, Falade-Nwulia O (2022). A longitudinal study of vaccine hesitancy attitudes and social influence as predictors of COVID-19 vaccine uptake in the US. Hum Vaccin Immunother.

[19] Berg MB, Lin L (2021). Predictors of COVID-19 vaccine intentions in the United States: the role of psychosocial health constructs and demographic factors. Transl Behav Med.

[20] McAfee T, Davis KC, Alexander RL, Pechacek TF, Bunnell R (2013). Effect of the first federally funded US antismoking national media campaign. Lancet.

[21] Farrelly MC, Duke JC, Nonnemaker J, MacMonegle AJ, Alexander TN, Zhao X, Delahanty JC, Rao P, Allen JA (2017). Association between the real cost media campaign and smoking initiation among youths - United States, 2014-2016. MMWR Morb Mortal Wkly Rep.

[22] Farrelly MC, Nonnemaker J, Davis KC, Hussin A (2009). The Influence of the National truth campaign on smoking initiation. Am J Prev Med.

[23] (2022). WHO coronavirus (COVID-19) dashboard. World Health Organization.

[24] (2022). COVID-19 vaccine effectiveness research. Centers for Disease Control and Prevention.

