# Supplementary material for: The Initial Relationship Between the United States Department of Health and Human Services’ Digital COVID-19 Public Education Campaign and Vaccine Uptake: Campaign Effectiveness Evaluation
Source: J Med Internet Res. 2023 May 3;25:e43873. doi: 10.2196/43873 (PMC10158813; doi:10.2196/43873)
Supplement: Multimedia Appendix 1 [file jmir_v25i1e43873_app1.docx]

**Multimedia Appendix**

Table of Contents

Additional Methods25

CABS Sample25

Paid Campaign Media Dose on Digital Platforms25

Date of COVID-19 Vaccine Uptake25

Covariates25

Survey Weighting25

Nielsen Digital Ad Ratings26

Table S1: Media Dose and Study Sample Sociodemographic Characteristics, United States Adults Ages 18 Years Old and Older April 1, 2021–November 16, 202127

Table S2: Unweighted Descriptive Statistics across Waves 1-328

Table S3: Model Controlling for Insurance Status29

Table S4: Categorical Demographic Model 30

**Additional Methods**

*CABS Sample*

Survey respondents were recruited through the National Opinion Research Center’s (NORC) AmeriSpeak probability-based research panel. Of the 4,398 respondents who completed the Wave 1 survey (administered from January 21 to February 2, 2021), 3,962 respondents completed the Wave 2 survey (administered from May 21 to June 29, 2021), and 3,642 respondents completed the Wave 3 survey (administered from September 23 to November 3, 2021). Wave-to-wave panel retention was high: 90%–92% of the previous wave’s respondents participated in each subsequent survey wave.

*Paid Campaign Media Dose on Digital Platforms*

Site direct, social media, and programmatic advertisements reflect the entirety of digital advertisements that can be purchased. Site direct advertisements are website advertisements that are purchased directly. Programmatic advertisements are purchased to reach audiences through an automated auction system in which many websites and apps participate. Social media advertisements are advertisements purchased on the social media platform and affiliated messaging platform and networks. In this study, these platforms included Facebook, Instagram, Twitter, Pinterest, Snapchat, and Reddit. Digital data were collected by National Opinion Research Center’s (NORC) at the University of Chicago’s AmeriSpeak probability-based research panel. For details on data collection see https://amerispeak.norc.org/us/en/amerispeak/about-amerispeak.html. Digital data are subject to restatement by digital publishers and social media platforms. This study used the best available digital data at the time of analysis.

*Date of COVID-19 Vaccine Uptake*

In Wave 3 of the survey, respondents were asked if they had received a COVID-19 vaccine (0 = *No, I have not received a COVID-19 vaccine* and 1 = *Yes, I have received at least one shot of a COVID-19 vaccine*). Those who reported having received at least one shot of a COVID-19 vaccine were asked to indicate the date on which they received the first vaccine shot by selecting the date, month, and year of receipt from drop-down menus.

*Covariates*

Cases and deaths data came from USAFacts.org,^25^ and cable news coverage of COVID-19 was derived from the Stanford Cable TV News Analyzer.^26^ Search terms of “COVID,” “COVID-19,” “coronavirus,” “Delta variant,” and “Omicron” were used to determine the total number of minutes COVID-19 was discussed by cable news outlets for a day.

Income was on a scale of 1 (*less than $50,000 per year*) to 4 (*greater than $100,000 per year*). A binary sex measure was used: male (1) and female (2). Age ranged from 1 (*18–24*) to 4 (*65+*). Education ranged from 1 (*no college*) to 3 (*bachelor’s degree*). Essential worker was a dichotomous measure with a value of 0 for non-essential workers and 1 for essential workers. Ideology ranged from 1 (*liberal*) to 3 (*conservative*). Preexisting health conditions was a dichotomous variable with a value of 0 indicating no health condition and 1 indicating at least one health condition.

Respondents were defined as “Vaccine Confident” if they indicated having received at least one dose of a COVID-19 vaccine or reported that they are “very likely” to get vaccinated. Respondents were defined as being in the “Movable Middle” if they reported that they are “somewhat likely,” “neither likely nor unlikely,” or “somewhat unlikely” to get vaccinated. Respondents were defined as “Vaccine Refusers” if they reported that they are “very unlikely” to get vaccinated.

*Survey Weighting*

All models were weighted and design adjusted (e.g., stratified, clustered) based on a series of adjustments from a panel weight provided by NORC (specific to the AmeriSpeak panel) that accounted for unequal sampling from the panel frame and unknown eligibility and nonresponse from eligible households. This household weight was calibrated using the U.S. Census Bureau’s Current Population Survey by sampling stratum to reflect the number of households per stratum. Subsequent adjustments to the survey weights accommodated person-level attributes, including nonresponse of adults within a household, as well as raking calibration adjustments by age, sex, race/ethnicity, age by sex, age by race/ethnicity, U.S. Census Division, education, household tenure, and household phone status (e.g., cell phone, landline). An additional adjustment was applied to the survey weights to accommodate nonresponse across waves 2 and 3 of the CABS to adjust for bias resulting from attrition.

The sampling strata were defined by age, race/ethnicity, education, and gender, resulting in 48 sampling strata. The survey weights were based on a series of adjustments that accounted for unequal sampling from the AmeriSpeak panel frame as well as unknown eligibility and nonresponse from eligible households. The unweighted AmeriSpeak panel does not precisely represent the overall U.S. population with respect to its demographic distribution. Unequal sampling accounts for differences between the AmeriSpeak panel composition and the overall U.S. population, while also accounting for minimal oversampling of select subgroups (e.g., by age and race/ethnicity) in Wave 1 to ensure that retention targets for each subgroup are reached in subsequent waves. DMAs were not a component of the sampling design, and the survey weights did not directly account for unequal sampling probabilities by DMA, given the differences in DMA population sizes. Rescaling the sampling weights has been shown in past research to bias the magnitude of variance components.^27^ We conducted sensitivity checks in which we rescaled the sampling weights to better accommodate between-DMA differences, which resulted in little change to the magnitude of our estimated coefficients and a more substantial (30%) decrease in the magnitude of the between-DMA variances. Because the effect of rescaling the sampling weights mainly affected the variance components, we did not adjust the weights in the primary model but note that the DMA-level variance in vaccination was likely an overestimate. Analyses did not adjust the change variable coefficients for known scaling bias, which is introduced when using non-repeated or absorbing outcomes, such as vaccination behavior.^28^ This bias can occur when most observations for the dependent variable are 0 and thus are not able to produce a meaningful difference score. Although such bias is necessarily introduced due to the nature of the dependent variable, the extent of this bias in the data is not necessarily problematic. In our sensitivity checks, we found that the results for digital impressions changed very little when applying the recommended correction using a linear regression-based instrumental variables estimator. We concluded that the scaling bias introduced by the non-repeated outcome was minimal; thus, we retained the linear mixed model as our estimator.

**Nielsen Digital Ad Ratings**

[Nielsen Digital Ad Ratings](https://gcc02.safelinks.protection.outlook.com/?url=https%3A%2F%2Fwww.nielsen.com%2Fsolutions%2Faudience-measurement%2Fdigital-ad-ratings%2F&data=05%7C01%7CSarah.Trigger%40hhs.gov%7Cabfc775b0c2d47d3b9df08da98131549%7Cd58addea50534a808499ba4d944910df%7C0%7C0%7C637989504976439040%7CUnknown%7CTWFpbGZsb3d8eyJWIjoiMC4wLjAwMDAiLCJQIjoiV2luMzIiLCJBTiI6Ik1haWwiLCJXVCI6Mn0%3D%7C3000%7C%7C%7C&sdata=b0KheHIHhYDZUB560xX4KVYjhMWjoVB%2FIMpptI5Oe0Q%3D&reserved=0) (DAR) delivers estimates of national, deduplicated audience reach and frequency of digital ad exposure across devices (computer, mobile, tablet, and connected TV). [Total Ad Ratings](https://gcc02.safelinks.protection.outlook.com/?url=https%3A%2F%2Fwww.nielsen.com%2Fsolutions%2Faudience-measurement%2Ftotal-ad-ratings%2F&data=05%7C01%7CSarah.Trigger%40hhs.gov%7Cabfc775b0c2d47d3b9df08da98131549%7Cd58addea50534a808499ba4d944910df%7C0%7C0%7C637989504976439040%7CUnknown%7CTWFpbGZsb3d8eyJWIjoiMC4wLjAwMDAiLCJQIjoiV2luMzIiLCJBTiI6Ik1haWwiLCJXVCI6Mn0%3D%7C3000%7C%7C%7C&sdata=NfsvoMKNjKzSEisHRHdKCh98myjK%2BwvMS1wlwapqF4o%3D&reserved=0) (TAR) further builds upon DAR estimates by combining DAR and Nielsen TV ratings to produce audience reach and frequency estimates across both traditional TV and digital channels. Nielsen’s proprietary approach includes adding tags to digital ads and audio watermarks to TV ads. For DAR, Nielsen leverages Facebook and other third-party data, census-based data, Nielsen panels, and advanced machine learning methodologies to translate impressions of tagged digital advertising at the individual level as part of Nielsen’s ID System. This system is calibrated using mobile surveys among Nielsen panel participants. This approach produces de-identified, people-based, cross-platform measurement of campaign reach and the average, per-person frequency of campaign exposure. Nielsen TAR adds an additional layer of TV campaign reach and frequency estimates using data from its National People Meter (NPM) TV ratings panel to provide a true cross-platform view of campaign performance across both TV and digital channels. For more information on Nielsen’s proprietary DAR and TAR tools, visit Nielsen.com. The HHS ASPA Campaign team commissioned a custom DAR and TAR study for the period of April 5, 2021–September 26, 2021, to validate internal estimates of Campaign reach and frequency; the final report was made on December 16, 2021.

**Table S1. Media Dose and Study Sample Sociodemographic Characteristics, United States Adults Ages 18 Years Old and Older April 1, 2021–November 16, 2021**

|  | Observations | Mean | Standard Deviation | Minimum | Maximum |
| --- | --- | --- | --- | --- | --- |
| Variables |  |  |  |  |  |
| DMA-Week-Person Varying |  |  |  |  |  |
| Week Vaccinated | 76,161 | 0.034 | 0.180 | 0.000 | 1.000 |
| DMA-by-Week Varying |  |  |  |  |  |
| Δ HHS Digital Impressions | 7,995 | 884.496 | 14,088.951 | -140,336.094 | 117,041.297 |
|  |  |  |  |  |  |
| Δ COVID-19 Cases per 100,000 people | 7,995 | 9.227 | 146.719 | -914.591 | 1,470.796 |
| Δ COVID-19 Deaths per 100,000 people | 7,995 | 3.856 | 4.097 | -10.744 | 96.365 |
|  |  |  |  |  |  |
|  |  |  |  |  |  |
| Week Varying |  |  |  |  |  |
| Δ COVID-19 Cable News Coverage | 47 | -1.622 | 20.218 | -60.218 | 49.393 |
| Person Varying |  |  |  |  |  |
| Wave 1 Vaccine Confidence | 3,431 | 2.298 | 0.727 | 1.000 | 3.000 |
| Income | 3,431 | 2.078 | 1.196 | 1.000 | 4.000 |
| Sex | 3,431 | 1.515 | 0.500 | 1.000 | 2.000 |
| Age | 3,431 | 2.674 | 0.923 | 1.000 | 4.000 |
| Education Level | 3,431 | 1.986 | 0.854 | 1.000 | 3.000 |
| Essential Worker | 3,431 | 0.321 | 0.467 | 0.000 | 1.000 |
| Ideology | 3,431 | 2.036 | 0.784 | 1.000 | 3.000 |
| Preexisting Health Condition | 3,431 | 0.645 | 0.478 | 0.000 | 1.000 |
| Rurality | 3,431 | 1.771 | 0.713 | 1.000 | 3.000 |
| Non-Hispanic Black | 3,431 | 0.120 | 0.325 | 0.000 | 1.000 |
| Hispanic/Latino | 3,431 | 0.168 | 0.374 | 0.000 | 1.000 |

*Note.* Due to small sample sizes, respondents who reported their race/ethnicity as American Indian or Alaska Native, non-Hispanic Asian American or Pacific Islanders, and non-Hispanic Multiple Race or Other Identity were omitted from the analysis and are thus not reported in this table.

**Table S2: Unweighted Descriptive Statistics Across Waves 1–3**

|  | Observations | Mean | Standard Deviation |
| --- | --- | --- | --- |
| Wave 1 Vaccine Confidence |  |  |  |
| Wave 1 | 4,398 | 2.284 | 0.750 |
| Wave 2 | 3,962 | 2.246 | 0.748 |
| Wave 3 | 3,642 | 2.296 | 0.732 |
| Income |  |  |  |
| Wave 1 | 4,208 | 2.103 | 1.203 |
| Wave 2 | 3,805 | 2.065 | 1.188 |
| Wave 3 | 3,506 | 2.072 | 1.194 |
| Sex |  |  |  |
| Wave 1 | 4,354 | 1.518 | 0.500 |
| Wave 2 | 3,925 | 1.521 | 0.500 |
| Wave 3 | 3,609 | 1.521 | 0.500 |
| Age |  |  |  |
| Wave 1 | 4,398 | 2.639 | 0.946 |
| Wave 2 | 3,962 | 2.638 | 0.944 |
| Wave 3 | 3,642 | 2.657 | 0.939 |
| Education Level |  |  |  |
| Wave 1 | 4,398 | 1.972 | 0.851 |
| Wave 2 | 3,962 | 1.975 | 0.850 |
| Wave 3 | 3,642 | 1.981 | 0.853 |
| Essential Worker |  |  |  |
| Wave 1 | 4,374 | 0.311 | 0.463 |
| Wave 2 | 3,943 | 0.314 | 0.464 |
| Wave 3 | 3,622 | 0.313 | 0.464 |
| Ideology |  |  |  |
| Wave 1 | 4,365 | 2.067 | 0.787 |
| Wave 2 | 3,949 | 2.073 | 0.784 |
| Wave 3 | 3,632 | 2.034 | 0.785 |
| Pre-existing Health Condition |  |  |  |
| Wave 1 | 4,371 | 0.633 | 0.482 |
| Wave 2 | 3,625 | 0.624 | 0.484 |
| Wave 3 | 3,633 | 0.640 | 0.480 |
| Rurality |  |  |  |
| Wave 1 | 4,383 | 1.787 | 0.720 |
| Wave 2 | 3,950 | 1.785 | 0.718 |
| Wave 3 | 3,632 | 1.765 | 0.712 |
| Black |  |  |  |
| Wave 1 | 4,070 | 0.131 | 0.338 |
| Wave 2 | 3,676 | 0.130 | 0.336 |
| Wave 3 | 3,376 | 0.129 | 0.336 |
| Hispanic |  |  |  |
| Wave 1 | 4,070 | 0.180 | 0.384 |
| Wave 2 | 3,676 | 0.180 | 0.384 |
| Wave 3 | 3,376 | 0.185 | 0.388 |

**Table S3. Model Controlling for Insurance Status**

|  | Model A1  (Std. Err.)  [P-Value] |
| --- | --- |
| Δ HHS Digital Impressions | 0.000013  (0.000004)  [0.002] |
| **Exogenous Factors** | |
| Δ COVID-19 Cases | -0.0006  (0.0003)  [0.064] |
| Δ COVID-19 Deaths | -0.0072  (0.0085)  [0.395] |
| Δ COVID-19 Cable News Coverage | 0.1577  (0.7275)  [0.828] |
| **Demographics** | |
| Income | 0.111  (0.028)  [0.000] |
| Female | -0.015  (0.062)  [0.813] |
| Age | 0.359  (0.039)  [0.000] |
| Education | 0.154  (0.041)  [0.000] |
| Essential Worker Status | -0.068  (0.083)  [0.409] |
| Political Ideology | -0.246  (0.043)  [0.000] |
| Preexisting Health Condition | 0.088  (0.054)  [0.102] |
| Rurality | -0.088  (0.049)  [0.076] |
| Black/African American | 0.064  (0.076)  [0.427] |
| Hispanic/Latino | 0.198  (0.076)  [0.009] |
| Insured | 0.302  (0.109)  [0.006] |
| **Initial Vaccine Confidence** | |
| Wave 1 Vaccine Confidence | 1.066  (0.058)  [0.000] |
| ***Week Dummy Variables Not Reported for Brevity*** | |
| Constant | -5.1973  (4.631)  [0.262] |
| DMA Variance | 0.104  (0.026) |
| Observations | 76,038 |
| DMAs | 204 |

*Note*. The dependent variable is a dichotomous measure of whether a respondent received the first dose of a COVID-19 vaccination in each week.

****p* < 0.001; ***p* < 0.01; **p* < 0.05

**Table S4. Categorical Demographic Model**

|  | Model A2  (Std. Err.)  [P-Value] |
| --- | --- |
| Δ HHS Digital Impressions | 0.000014  (0.000004)  [0.001] |
| **Exogenous Factors** | |
| Δ COVID-19 Cases | -0.0006  (0.0003)  [0.052] |
| Δ COVID-19 Deaths | -0.0083  (0.0083)  [0.320] |
| Δ COVID-19 Cable News Coverage | 0.1253  (0.7289)  [0.864] |
| **Demographics** | |
| Income  50,000–75,000  75,000–100,000  Greater than 100,000 | 0.302  (0.074)  [0.000]  0.218  (0.076)  [0.004]  0.457  (0.085)  [0.000] |
| Female | 0.005  (0.064)  [0.936] |
| Age  25–44    45–64    65+ | -0.314  (0.120)  [0.009]  -0.156  (0.116)  [0.171]  0.878  (0.122)  [0.000] |
| Education  Some College    Bachelor’s Degree or Higher | 0.141  (0.0702)  [0.045]  0.436  (0.077)  [0.000] |
| Essential Worker Status | 0.033  (0.083)  [0.691] |
| Political Ideology  Moderate  Conservative | -0.178  (0.069)  [0.010]  -0.520  (0.879)  [0.000] |
| Preexisting Health Condition | 0.113  (0.051)  [0.028] |
| Rurality  Suburban  Rural | 0.012  (0.078)  [0.875]  -0.242  (0.102)  [0.017] |
| Black/African American | 0.049  (0.078)  [0.531] |
| Hispanic/Latino | 0.192  (0.080)  [0.017] |
| **Initial Vaccine Confidence** | |
| Wave 1 Vaccine Confidence | 1.082  (0.060)  [0.000] |
| ***Week Dummy Variables Not Reported for Brevity*** | |
| Constant | -4.445  (4.630)  [0.337] |
| DMA Variance | 0.102  (0.028) |
| Observations | 76,128 |
| DMAs | 204 |

*Note*. The dependent variable is a dichotomous measure of whether a respondent received the first dose of a COVID-19 vaccination in each week.

****p* < 0.001; ***p* < 0.01; **p* < 0.05
